# Advances in high‐throughput mass spectrometry in drug discovery

**DOI:** 10.15252/emmm.202114850

**Published:** 2022-12-14

**Authors:** Maria Emilia Dueñas, Rachel E Peltier‐Heap, Melanie Leveridge, Roland S Annan, Frank H Büttner, Matthias Trost

**Affiliations:** ^1^ Laboratory for Biomedical Mass Spectrometry, Biosciences Institute Newcastle University Newcastle‐upon‐Tyne UK; ^2^ Discovery Analytical, Screening Profiling and Mechanistic Biology, GSK R&D Stevenage UK; ^3^ Drug Discovery Sciences, High Throughput Biology Boehringer Ingelheim Pharma GmbH&CoKG Biberach Germany

**Keywords:** affinity selection, drug discovery, high‐throughput screening, MALDI‐TOF, mass spectrometry, Methods & Resources, Pharmacology & Drug Discovery

## Abstract

High‐throughput (HT) screening drug discovery, during which thousands or millions of compounds are screened, remains the key methodology for identifying active chemical matter in early drug discovery pipelines. Recent technological developments in mass spectrometry (MS) and automation have revolutionized the application of MS for use in HT screens. These methods allow the targeting of unlabelled biomolecules in HT assays, thereby expanding the breadth of targets for which HT assays can be developed compared to traditional approaches. Moreover, these label‐free MS assays are often cheaper, faster, and more physiologically relevant than competing assay technologies. In this review, we will describe current MS techniques used in drug discovery and explain their advantages and disadvantages. We will highlight the power of mass spectrometry in label‐free *in vitro* assays, and its application for setting up multiplexed cellular phenotypic assays, providing an exciting new tool for screening compounds in cell lines, and even primary cells. Finally, we will give an outlook on how technological advances will increase the future use and the capabilities of mass spectrometry in drug discovery.

## Graphical abstract

This Review summarizes advantages and disadvantages of high‐throughput mass spectrometry techniques used in drug discovery and discusses how technological advances could increase the capabilities of mass spectrometry in drug discovery in the future.

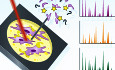

**Glossary**
BLAZE mode: The name of the RapidFire hardware modification that improves the speed of the system by enabling cycling times of 2.5 s per sample
Chemoproteomics: A broad set of techniques used to identify and characterize the mode of action of a drug. This can include quantitative MS‐based proteomics
Data‐independent acquisition MS: A recently developed global MS‐based proteomics strategy that first isolates precursor ions into pre‐defined isolation windows, which are then fragmented and analysed
Fragment‐based drug discovery: Method used to develop potent small‐molecule compounds starting from fragments binding weakly to targets
Limited proteolysis MS: Used to measure protein structural transitions directly in biological matrices and on a proteome‐wide scale
Mechanism of action: Refers to the specific biochemical interaction through which a drug substance produces its pharmacological effect
PhAbit: PhotoAffinity bits. A reversible ligand with a photoreactive warhead incorporated to facilitate covalent binding
Phosphoproteomics: Proteomics analysis that seeks to determine the overall level of protein phosphorylation and the identity of proteins, which are phosphorylated, and amino acid residues, which hold the phosphate group
RapidFire: Is a proprietary automated microfluidic sample collection and purification system that interfaces directly to standard ESI‐MS instruments. This system uses high‐speed robotics to directly aspirate fluidic samples from 96‐ or 384‐well screening plates, rapidly removes non‐volatile assay components such as salts, buffers and detergents in an online fractionation step, and delivers purified analytes to the mass spectrometer
Size exclusion chromatography: A chromatographic separation technique that separates analytes by size, and, therefore, relative to molecular weight
Thermal proteome profiling: A quantitative MS‐based proteomics tool used to monitor the melting profile of thousands of proteins simultaneously
Warhead: A reactive group that is strategically incorporated onto a reversible ligand to facilitate the formation of a covalent bond to a target biomolecule

## Introduction

The drug discovery and development pipeline is an interdisciplinary process that engages multiple phases of research to facilitate the generation of effective therapies (Mohs & Greig, 2017). The historical aspects of the traditional drug discovery pipeline have been extensively reviewed and demonstrate the advantages and challenges of drug discovery throughout R&D including productivity, attrition, and evolution of new technologies (Moffat *et al*, 2017; Vincent *et al*, 2022). The drug discovery phase contains the target identification and validation phase, as well as hit finding, typically through high‐throughput screening (HTS) campaigns employing large compound libraries of several hundred thousands of compounds. At the end of this phase, chemistry is performed to optimize the activity and physicochemical properties of the molecule, both of which influence its *in vivo* behavior as it relates to potency, clearance, and safety. Early adoption of new technologies can be critical to improving R&D as there are often lengthy cycle times and high failure rates of drug discovery projects prior to pre‐clinical development. There is, therefore, a focus across industry and academia on the development of more biologically relevant and diverse approaches to the discovery of chemical starting points, to address both the success rates and pace of research.

Mass spectrometry (MS) is a powerful, versatile technique with applications spanning the full spectrum of the drug discovery and development pipeline. For example, MS techniques such as proteomics, metabolomics and analysis of clinical tissue samples are an important part of target validation, as well as later in discovery where these techniques can be used to gain insight into a compound's cellular mechanism of action (MoA). During lead optimization, MS has for decades played the central role in determining both the structure and pharmacokinetic properties of compounds. MS is also increasingly important in the target identification step of the drug discovery pipeline. For example, limited proteolysis‐coupled MS (Schopper *et al*, 2017) is routinely used to determine proteome‐wide specificity and uncover small molecule binding sites, thermal proteome profiling (Franken *et al*, 2015) for small molecule target finding, and data‐independent acquisition MS for HT analysis of cell systems for global proteomics and phosphoproteomics (Kitata *et al*, 2021).

Despite MS being a powerful tool within the overall drug discovery process, its application to HT screening has lagged, often due to a lack of throughput and lack of associated automation. Current HTS assays are often performed using fluorescence and chemiluminescence‐based detection modalities that although HT, are susceptible to compound‐dependent screening artefacts leading to false positives or negatives (Winter *et al*, 2018). Here, MS presents itself as an attractive alternative technology as it is already an established, sensitive, and versatile technique in research for the analysis of small and large biomolecules. A key advantage of MS has been the potential to build label‐free assays that improve hit confirmation rates and ultimately accelerate the drug discovery process. HTS‐MS has been demonstrated to be an effective tool for removing potential detection‐based false positives and thus mitigating sources of assay interference (Adam *et al*, 2015). From orthogonal to traditional hit‐finding approaches, MS presents the opportunity to explore alternative hit‐identification strategies that focus on detecting protein‐target binders, or compounds that directly modulate cellular function to reverse or treat a disease phenotype.

The aim of this review is to provide an overview of the recent developments in HT‐MS for drug discovery. We outline how these advancements in MS have enabled the development of HTS‐MS platforms and their applications. Finally, we provide an outlook of how technological advances could further drive alternative capabilities of MS in drug discovery.

## Basic principles of mass spectrometry instrumentation

MS is an analytical technique that measures both the mass‐to‐charge ratio (*m/z*) and abundance of ions to generate a mass spectrum that can in turn yield chemically relevant information such as empirical mass or structure about a particular analyte. In its simplest form, a mass spectrometer consists of an ionization source coupled to a mass analyzer and detector. The ion source transfers sample molecules into the gas phase as charged ions which then are transferred into a mass analyzer. Here, ions are separated based on their *m/z* and detected, thus generating a mass spectrum. As not only the *m/z* but also the number of detected ions is recorded, MS can be a highly quantitative technique with a linear range of up to ~10^5^ (Collings *et al*, 2014).

**Figure 1:**
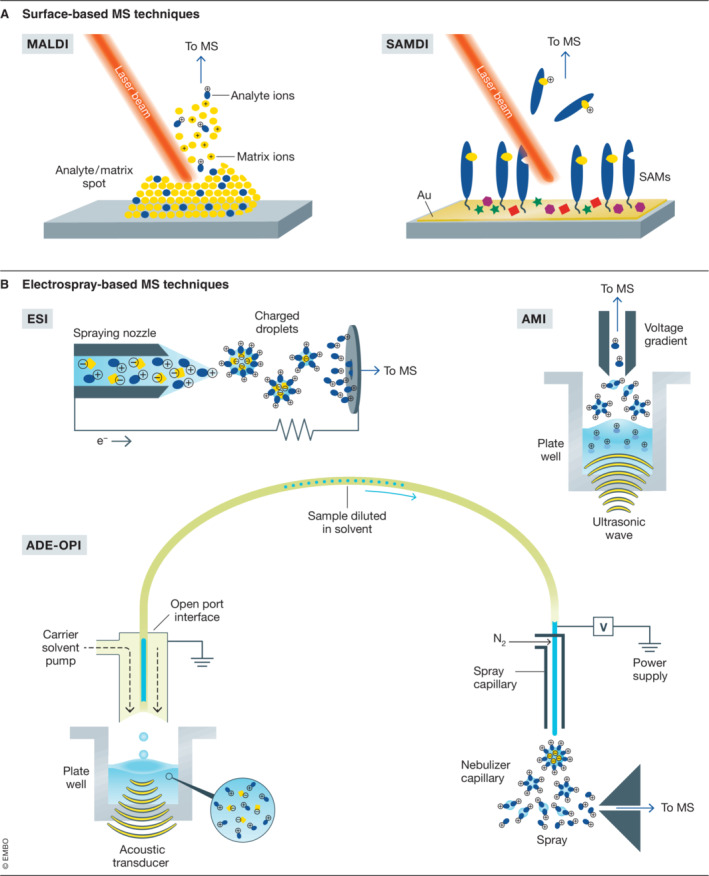
Schematic of main ionization techniques employed for HTS‐MS (A) Surface‐based: MALDI. Samples are co‐crystallized with a matrix on a conductive target plate. Laser shots are used to activate matrix molecules and evaporate analyte and matrix. In the reactive cloud, protons are transferred from the matrix to ionize the analyte molecules (Karas *et al*, 1985). SAMDI. Components of an enzymatic reaction (either enzymes or substrates) are immobilized onto self‐assembled monolayers (SAMs) in an array format, and upon irradiation with a laser, the monolayers are desorbed from the surface through cleavage of the thiolate‐gold bond and ionized (Gurard‐Levin *et al*, 2011). (B) Electrospray‐based: ESI. The analytes are dissolved in a liquid carrier phase, and a high voltage is applied to the tip of the metal capillary relative to the mass spectrometer's sampling cone. The electric field causes the dispersion of the sample solution resulting in nebulization. Charged droplets containing the analytes are generated at the exit of the electrospray tip. The solvent of the droplets is vaporized by a drying gas or heat and the charged analytes are guided by a potential gradient toward the analyzer region of the MS (Fenn *et al*, 1989; El‐Aneed *et al*, 2009). AMI. An acoustic transducer and charging cone are used to generate nanolitre‐sized charged droplets that are guided through an ion transfer line into a MS (Sinclair *et al*, 2015). ADE‐OPI. A pulse of acoustic energy ejects sample droplets upward into the inverted OPI, where a fluid pump delivers carrier solvent to a sample capture region. The sample is captured, diluted, and guided to MS by conventional ESI (Zhang *et al*, 2021).

HT‐MS‐based readouts in drug discovery have been largely dominated by instruments comprising of solid‐phase extraction (SPE) coupled to electrospray ionization (ESI), or surface‐based techniques such as matrix‐assisted laser/desorption ionization (MALDI). Self‐assembled monolayers (SAMs) coupled with desorption/ionization (SAMDI), as well as some more recent approaches such as acoustic mist ionization (AMI), and acoustic droplet ejection (ADE) open port interface (OPI) MS have been added to the toolbox. These principles are described in Fig 1 (surface‐based, Fig 1A and electrospray‐based Fig 1B). Each of these ionization techniques can be combined with different mass analyzers to access different levels of mass resolution, dynamic ranges, analysis time, and sample throughput. For a detailed review, please see Challen and Cramer (2022).

## Mass spectrometry screening assays for drug discovery

### Biochemical and functional assays to identify inhibitors of enzymes

**Figure 2:**
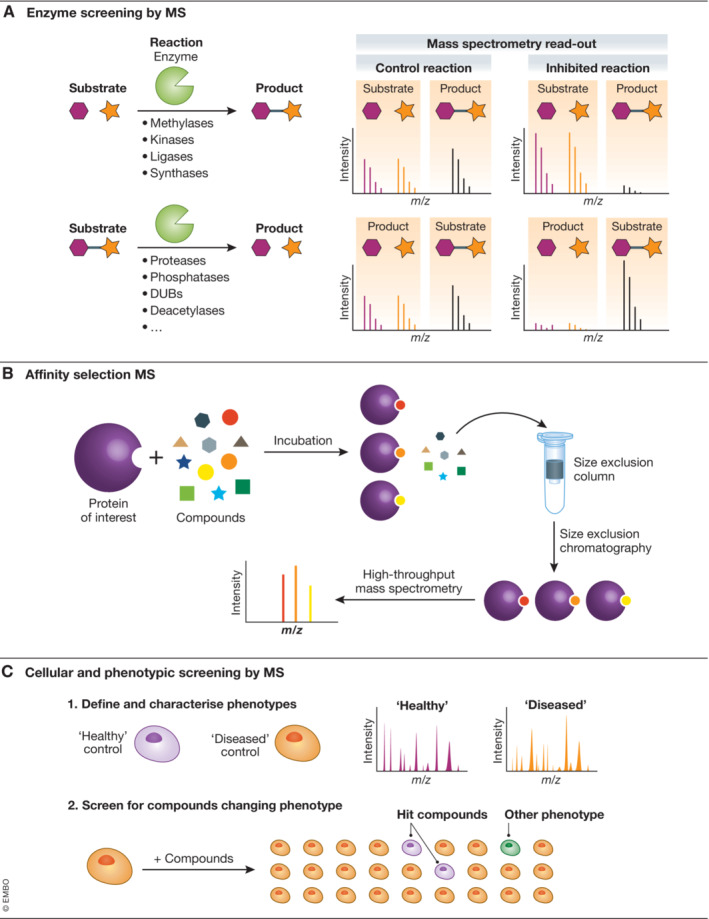
Types of high‐throughput mass spectrometry drug discovery assays (A) Enzyme activity screening by mass spectrometry. *In vitro* reactions of enzymes with substrates are stopped at appropriate time points and the resulting mixture analysed by mass spectrometry to identify substrate to product conversion. Addition of chemical compounds that affect the reaction are identified by reduced product conversion. (B) Affinity Selection Mass Spectrometry. Compounds bind to a protein of interest and non‐binding compounds are removed by size‐exclusion chromatography. Binding compounds are identified by mass spectrometry. (C) Cellular and phenotypic screening by mass spectrometry. Cellular phenotypes of “healthy” and “diseased” controls are defined by a read‐out of a cellular “fingerprint” of specific biomolecules. Chemical compounds that shift the “diseased” phenotype to “healthy” are considered hits.

Once target proteins have been identified as a potential drug target in a specific disease, biochemical *in vitro* assays are often performed to identify molecules that modulate protein function. For protein targets that are enzymes, target inhibition or activation can be measured via the generation of a product, or the decrease of a substrate, in a biochemical reaction (Fig 2A). Unlike most traditional biochemical assays, MS allows the direct, label‐free quantitative measurement of both substrate and product in these *in vitro* assays, as long as a mass shift occurs; therefore, most enzyme targets are principally amenable for mass spectrometric analysis. In recent years, ion mobility separation has been integrated within new HTS capable mass spectrometers, thus enabling the separation of complex and isobaric compounds such as lipid classes (Djambazova *et al*, 2020). This will likely broaden the development of HTS‐compatible MS assays for challenging enzymes, such as isomerases, in future years.

For ESI, different technologies such as the RapidFire system in BLAZE‐mode (Bretschneider *et al*, 2019) or ADE‐OPI MS approach have been described for enzymatic‐type assays (Häbe *et al*, 2020; Simon *et al*, 2021a). The versatility of the instrument setup allows the analysis of many different biomolecules including lipids (Highkin *et al*, 2011; Dittakavi *et al*, 2020), peptides (Hutchinson *et al*, 2011; Liddle *et al*, 2020), and metabolites (Soulard *et al*, 2008; Maxine *et al*, 2009), from a wide range of matrix systems including blood, plasma (Highkin *et al*, 2011; Bretschneider *et al*, 2017) and cell lysates (Gordon *et al*, 2016; Dittakavi *et al*, 2020). Ambient ionization, such as desorption electrospray ionization (DESI), which commonly does not require sample preparation, is a new and attractive alternative for HT analysis. DESI‐MS displays remarkably high salt tolerance, making this technique ideal for the analysis of complex samples without any sample preparation. Using DESI, samples are ionized outside of a mass spectrometer under native conditions. Due to its ability to rapidly scan a surface, DESI‐MS has been amenable to HT applications (Wleklinski *et al*, 2018), at rates approaching 10,000 reactions per hour, and for the analysis of enzymatic reactions directly from the bioassay matrix (Morato *et al*, 2020).

The development of instrumentation and improvements in sample preparation have enabled MALDI‐ time‐of‐flight (TOF) MS to rival the more conventional HTS assays with throughputs of 10–20 samples per second for conventional MALDI (Haslam *et al*, 2015) and liquid atmospheric pressure MALDI (Krenkel *et al*, 2022) reported. The first drug discovery studies using the high speed (1,536 spots in less than 8 min) of these new‐generation MALDI‐TOF mass spectrometers for drug discovery was the development of a HTS compatible assay to study the specificity and drugability of deubiquitylases (DUBs; Ritorto *et al*, 2014). In this work, individual DUBs were incubated with ubiquitin dimers of different linkage type and the quantitation of mono‐ubiquitin using an isotopically labelled internal standard enabled the determination of DUB specificity, and this was further applied to drug screening. This assay was unique to the field as it used native substrates, rather than the previously used rhodamine fluorescently labelled reagents (Hassiepen *et al*, 2007), and also had the potential to be expanded to a HT drug screening platform.

HT MALDI‐TOF MS assays targeting post‐translational modifications have grown rapidly in the past decade as the technique can be applied to potentially any reaction that involves a mass change. This importantly allows label‐free quantitation, a gold standard for assays in the drug discovery field with respect to simplicity and cost. Successful MALDI‐TOF MS assays now include the study of kinases (Beeman *et al*, 2017; Heap *et al*, 2017), methyltransferases (Guitot *et al*, 2017), and phosphatases (Winter *et al*, 2018).

Most of the MALDI‐TOF‐based HTS‐compatible approaches conducted so far have focused on *in vitro* assays with simple readouts (with often just a single substrate and product) and have been limited to peptide/protein‐centric activity assays (Ritorto *et al*, 2014; Guitot *et al*, 2017; Heap *et al*, 2017; De Cesare *et al*, 2018; Winter *et al*, 2018; Simon *et al*, 2020). Applying this technology for cellular assays and metabolomics‐based drug discovery remains a challenge mostly due to (i) interference from matrix peaks in the low‐mass range, (ii) matrix‐dependent analyte selectivity, and (iii) limited metabolite coverage due to low sensitivity of certain classes of metabolites. Although, recently, individual metabolites such as trimethylamine (Winter *et al*, 2019), acetylcholine (Chandler *et al*, 2016), 3‐methoxytyramine (Winter *et al*, 2022), and cyclic GMP‐AMP (at a throughput of ~60,000 samples per day; Simon *et al*, 2020) have been used in MALDI‐TOF HTS campaigns, new tools and methods need to be developed to meet the opportunities and challenges toward HT metabolic profiling for drug discovery.

The SAMDI technology is a promising strategy for HTS that uses the same MALDI‐TOF MS instrumentation but in a more targeted approach where immobilized proteins are used to capture substrates or products (Gurard‐Levin *et al*, 2011). Although generally not label‐free, as the protein needs to have a tag to be immobilized, this technology enables the specific capture of analytes and is well suited for measuring a broad range of enzyme activities as SAMs can be customized to use a variety of immobilization chemistries (Mrksich, 2008). An exemption to this statement is traceless‐SAMDI (Helal *et al*, 2018). This work introduced a truly label‐free approach for analysing HT reactions by using a photogenerated carbene to non‐selectively attach molecules to the SAMs, from which can then be analyzed by MS. SAMDI has also been used for *in vitro* recombinant enzyme/substrate screen on diverse enzyme classes, such as methyltransferases (Swalm Brooke *et al*, 2013), glycosyltransferases (Ban *et al*, 2012), and deacetylases (Gurard‐Levin *et al*, 2010). Selected publications describing HTS compatible MS assays can be found in Table 1.

**Table 1:** Selected publications describing HTS MS‐compatible assays in drug discovery.

Enzyme	Substrate	Product	Platform	Citation
Phosphatidylserine decarboxylase	Phosphatidylserine	Phosphatidylethanolamine	RapidFire	Forbes *et al* (2007)
ERAP1	Peptide	Peptide	RapidFire	Liddle *et al* (2020)
Acetyl‐coenzyme A carboxylase	Sphingosine in whole blood	Sphingosine‐1‐phosphate	RapidFire	Maxine *et al* (2009)
Autotaxin	Lysophosphatidyl choline	Lysophosphatidic acid	RapidFire	Soulard *et al* (2008)
Histone lysine demethylase	Trimethylated peptide	Demethylated peptide	RapidFire	Hutchinson *et al* (2011)
Histone deacetylase	Acetylated peptide	Peptide	AMI‐MS	Sinclair *et al* (2019)
Histone acetyltransferase	Peptide and acetyl‐CoA cofactor	Acetylated peptide	AMI‐MS	Belov *et al* (2020)
Diacylglycerol acyltransferase 2	Diolein and oleoyl‐CoA	triolein	ADE‐OPI MS	Wen *et al* (2021)
Cyclic GMP‐AMP synthase	GTP + ATP	Cyclic GMP‐AMP	ADE‐OPI MS	Simon *et al* (2020)
Deubiquitylases	Diubiquitin	Ubiquitin	MALDI‐TOF	Ritorto *et al* (2014)
E3‐ligases	Diubiquitin	Ubiquitin	MALDI‐TOF	De Cesare *et al* (2018) and De Cesare *et al* (2020)
Kinases	Peptide	Phosphopeptide	MALDI‐TOF	Beeman *et al* (2017) and Heap *et al* (2017)
Methyltransferases	Peptide	Methylated peptide	MALDI‐TOF	Guitot *et al* (2017), Guitot *et al* (2014) and Haslam *et al* (2015)
Phosphatases	Phosphopeptide	Peptide	MALDI‐TOF	Winter *et al* (2018)
Acetylcholinesterase	Acetylcholine	Choline	MALDI‐TOF	Haslam *et al* (2015)
Cyclic GMP‐AMP synthase	GTP + ATP	Cyclic GMP‐AMP	MALDI‐TOF	Simon *et al* (2020)
Anthrax lethal factor	Peptide	Peptide	SAMDI	Min *et al* (2004)
Sirtuin 3	Acetylated peptide	Peptide	SAMDI	Patel *et al* (2015)
Methyltransferases	Peptide	Methylated peptide	SAMDI	Swalm Brooke *et al* (2013)
Glycosyltransferases	Saccharides	Oligosaccharides	SAMDI	Ban *et al* (2012)
Deacetylases	Acetylated peptide	Peptide	SAMDI	Gurard‐Levin *et al* (2010)
Isocitrate dehydrogenase 1	Isocitrate	α‐ketoglutarate	MALDI + ESI	Radosevich *et al* (2022)
Catechol‐O‐methyltransferase	Dopamine	3‐methoxytyramine	MALDI‐TOF	Winter *et al* (2022)

### Affinity and binding assays

Affinity selection mass spectrometry (ASMS) is a HT and cost‐effective binding assay that enables rapid screening of a large number of compounds against a specific target biomolecule of interest (Prudent *et al*, 2021). In a traditional HT ASMS approach (Fig 2B), the biomolecular target is typically present in molar excess relative to the potential ligands that are then captured by the protein. Non‐bound ligands are separated from the protein using usually either an affinity enrichment or size exclusion chromatography (SEC). Bound ligands are then dissociated from the target protein and identified by their accurate mass with a suitable MS technique. Alternatively, ASMS can also be employed as an assay to further characterize ligand‐binding properties, such as to demonstrate proof of binding as well as performing competition experiments (Simon *et al*, 2021b). ASMS has emerged over the past two decades as a strategy complimentary to functional HTS assays (Annis *et al*, 2007). This approach leverages the label‐free and direct detection capability of MS and is most often coupled to SEC. In particular, it has been widely adopted in industry due to its scalability and led to the development of fully automated systems, such as the Automated Ligand Identification System (Annis *et al*, 2004), as well as the SpeedScreen system (Muckenschnabel *et al*, 2004; Zehender *et al*, 2004; Zehender & Mayr, 2007). Typically, a 1 million compound screen with a pooling strategy can take 5–7 days with follow‐up experiments ranging 1–3 weeks to re‐confirm and characterize compound binding depending on the strategy employed. The rationale behind the affinity selection approach is that binding must precede activity, therefore, the identification of small molecule binders can be a surrogate to reading out activity in a traditional HT biochemical assay during the first stages of a hit ID campaign. Advantageously, this can identify ligands that exhibit multiple MoA, potentially identifying agonists and antagonists in a single screen. An ASMS HTS can often be less complex to develop than a traditional biochemical HTS and can accommodate targets where very little knowledge of protein function or structure exists. By designing ASMS specific collections or mass encoded libraries, a broader screening of chemical space could be possible to reduce complex downstream deconvolution and redundancies. (Prudent *et al*, 2021).

MS has been instrumental in the development of ASMS strategies and HT screens of more than one million compounds have been achieved across in‐solution ASMS platforms. These include a diverse range of targets like beta‐secretase (Coburn *et al*, 2004), G‐protein coupled receptors (Whitehurst & Annis, 2008), RNA polymerase (Walker *et al*, 2017), CHK1 (Comess *et al*, 2006) and to probe druggable target space within the NF‐kβ pathway (Kutilek *et al*, 2016). These screens have historically been performed using pools of 100–2,000 compounds and analysis on high‐resolution MS instruments. This approach, although HT, does suffer a few analytical challenges. Typically, protein concentrations in the micromolar range are needed and good protein solubility over 12–24 h is critical, which can be problematic for some targets like membrane proteins. Furthermore, the use of large pools of compounds can increase overall DMSO concentration, reduce assay sensitivity, and could also denature the target protein structure. More recently, HTS capable MALDI‐TOF MS platforms that use faster instrument scanning speeds have been used to screen smaller pools of compounds by ASMS. This includes the SEC MALDI‐TOF MS platform proposed by Simon *et al* (2021b), as well as a SAMDI‐TOF MS approach, both of which use pools of tens of compounds rather than hundreds, yet can still reach the same sample throughput.

### Covalent fragment assays in drug discovery

Fragment‐based drug discovery (FBDD) is an established, versatile strategy in drug discovery that aims to develop novel drugs from small, low molecular weight starting points. Sensitive technologies, including surface plasmon resonance, nuclear magnetic resonance, and MS, have been used to detect the binding or activity of these fragments. An excellent example of this approach is the discovery of vemurafenib, a selective inhibitor of the oncogenic target B‐RAF (Tsai *et al*, 2008). Advantages of FBDD often include reduced experimental costs, as well as novel strategies to developing new drugs that harness advances in HT chemistry.

One aspect of FBDD where MS technology has been instrumental is the development of reactive or covalent fragment screening strategies. This approach exploits the advances made in synthesis of small molecule libraries that can then be coupled to covalent warheads to accelerate screening efforts (Lu *et al*, 2021). Using small covalent fragments to probe biological systems and poorly characterized targets significantly enhances our ability to translate traditional biological research to the development of new medicines and understanding their MoA (Schreiber Stuart *et al*, 2015; Zhang *et al*, 2019). This has been particularly impactful where probes were used to explore the therapeutic effects of PKM2 activation in cancer (Anastasiou *et al*, 2012; Kung *et al*, 2012), and in the generation of a covalent inhibitor of KRAS that was previously thought to be undruggable (Naim *et al*, 2021). Novel chemotypes for anti‐malarial therapeutics have also been described, along with their MoA, through use of covalent probes (Heidebrecht *et al*, 2012); this in turn may infer on future paths of resistance (Lukens *et al*, 2015).

With this screening approach, an irreversible binding event of the fragment to a protein is observed. MS plays a key role in the screening of these compounds as the addition of the fragment molecule induces a shift in the protein molecular weight that can then be measured by MS. These MS techniques can then support the characterization of a compounds MoA as well as identify specific protein target engagement. The binding event itself is highly dependent on the synergistic relationship between structural biology and synthetic chemistry to enable binding to a relevant site of interest and often yields key mechanistic insights. In comparison to the use of a traditional screening collection, it has been shown that a fragment‐based screening approach can offer better coverage of chemical space, as well as identify novel chemical equity that interacts with a protein binding pocket (Hall *et al*, 2014). Furthermore, this approach has been particularly successful for studying targets where traditional compound collections have been unsuccessful (Coyne *et al*, 2010).

**Figure 3:**
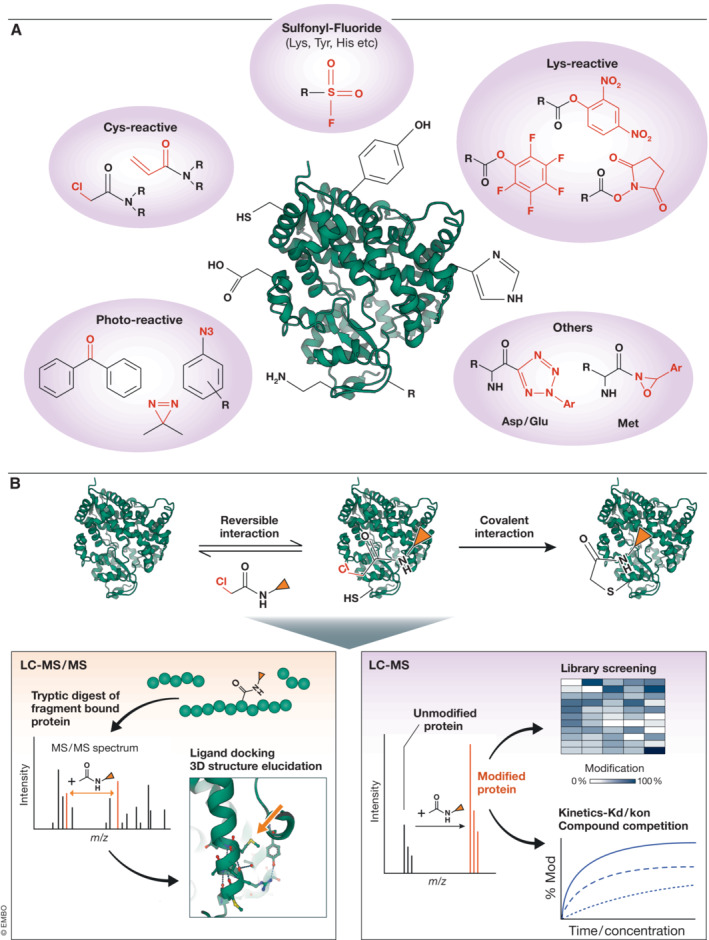
Fragment‐based drug discovery assays (A) Summary of amino acid targeting moieties with the reactive warheads highlighted in red. (B) Overview of a reactive fragment covalently binding to a protein target of interest and how LC–MS and LC–MS/MS contribute to the measurement of binding affinity, localization of covalently bound residue as well as kinetic and competition studies.

Perhaps the simplest reactive FBDD approach is the screening of fragment libraries against recombinant target proteins *in vitro*. Here, low molecular weight (typically < 300 Da) fragment libraries that typically contain electrophilic properties are synthesized and coupled to covalent warheads (Long & Aye, 2017). These libraries are then screened against a specific target protein and hits are distinguished by liquid chromatography (LC)‐MS (Fig 3). These libraries have historically been relatively small in the orders of 100–1000s of compounds and thus do not require the use of uHTS mass spectrometers. A LC–MS or RapidFire MS approach to covalent fragment screening is often in the order of 0.5–5 min per sample. Many studies have used this technology to screen fragments against various targets such as the E3 ligase HOIP (Johansson *et al*, 2019), BRD4 (Grant *et al*, 2019; Olp *et al*, 2020), GDP‐KRAS^G12C^ (Shin *et al*, 2019), and OTUB2 and NUDT7 (Resnick *et al*, 2019). Selected publications of fragment‐based MS assays can be found in Table 2.

**Table 2:** Selected publications of fragment‐based MS screening assays.

Reactive fragment	Warhead	Target protein	MS platform	Citation
Cys‐reactive	Acrylate	Pappain	LC‐TOF	Kathman *et al* (2014)
Cys‐reactive	α,β‐unsaturated methyl ester	HOIP	LC‐TOF	Johansson *et al* (2019)
Cys‐reactive	Chloroacetamide	Pin1	LC‐TOF	Dubiella *et al* (2021)
Cys‐reactive	Acrylate	BRD domains	LC‐Ion Trap	Olp *et al* (2020)
Cys‐reactive	Acrylamide	KRas/KRas(G12C)	LC‐TOF	Ostrem *et al* (2013)
Lys‐reactive	Acrylate/alpha‐beta unsaturated esters	HSP72	LC‐qTOF and LC–MS/MS	Pettinger *et al* (2017)
Lys‐reactive	Aryl boronic acid carbonyl	Mcl‐1	LC‐TOF	Akçay *et al* (2016)
PhABit	Alkyl diazirine/alkyne tag	BRD4/KRas	RapidFire‐TOF	Grant *et al* (2020)
PhABit	5× photoreactive groups	CDK(2/7/9)	LC‐TOF	Grant *et al* (2019)
PhABit	Diazirine	CA(ii)	RapidFire‐TOF	Thomas *et al* (2021)
Tyr‐reactive	Sulfonyl‐fluoride	GST's	LC–MS/MS	Shishido *et al* (2017)
Ser‐reactive	Aryl fluorosulfate	DcpS	LC‐TOF/LC–MS/MS	Fadeyi *et al* (2017)

As covalent fragment screening is still a relatively new approach, a number of challenges do hinder its potential to come to the forefront of drug discovery. For example, although warhead design is expanding to target a range of amino acids in active site pockets (Fig 3A), their selectivity can be limited and sometimes the reactivity of the warhead rather than fragment affinity can drive binding. Furthermore, some of the more reactive warheads currently used in screening, such as chloroacetamides, are not always suitable for translating into the clinic due to toxicity. Finally, current LC–MS approaches used for covalent fragment screening (Fig 3B) lack the throughput of other technologies discussed in this review such as MALDI and ADE. Advances in future MS instrumentation, such as ion mobility, may support the implementation of covalent FBDD in routine drug discovery efforts.

With MS underpinning the majority of chemical biology approaches to covalent drug discovery and chemoproteomics, many of the advances made in this field have been made hand‐in‐hand with the development of newer, faster, and higher resolution MS instruments. It is expected that chemical biology will continue to be a major driver of modern drug discovery approaches and understanding of the illusive, yet potentially therapeutic human proteome.

### Moving toward cellular and phenotypic MS drug discovery assays

Phenotypic screening is common for target identification as a result of genetic perturbation of a biological system such as CRISPR edits and chemogenomic screening (Jones & Bunnage, 2017; Jost & Weissman, 2018). In some cases, phenotypic screening can also be used orthogonally to identify and validate molecules that alter a specific *in vitro* cellular phenotype in a mechanistically agnostic manner (Fig 2C). This approach can identify compounds that are active against multiple targets and unknown pathways in physiologically relevant disease models. Until recently, microscopy‐based approaches have been the most common way to read out parameters of the cellular phenotype and the potential shift toward a desired phenotype. Recently, there has been interest to explore MS approaches to unbiasedly screen the phenotype by monitoring specific metabolites, protein substrates and activation of biological pathways.

While MS‐based *in vitro* HTS assays are achievable on current instrumentation, there is a renewed interest in the pharmaceutical industry toward phenotypic cellular assays as these allow the identification of novel pathways that lead to the wanted outcome; most of these assays are currently performed using fluorescence microscopy or flow cytometry. Cellular assays for evaluating compound efficacy at moderating or reversing a cellular phenotype presents an interesting challenge for MS platforms as the system becomes inherently more complex.

A well‐established application for whole cell phenotyping is the classification of micro‐organisms by MALDI‐TOF MS, also known as biotyping (Claydon *et al*, 1996). Throughout the past decades, this has led to sensitive, robust, and inexpensive phenotyping of microorganisms (Mutters *et al*, 2014; Patel, 2015). Application of whole cell MALDI‐TOF MS methodologies to mammalian cells has not yet reached the heights of typical microbial biotyping methods but is rising as a promising technology for phenotypic screening and development of drug discovery assays. Compared to microbial cells, eukaryotic cells have a much higher complexity, with intricate cellular networks and cell cycle states influenced by their spatial anatomy (Munteanu & Hopf, 2013), making them an excellent target for MALDI‐TOF MS.

**Figure 4:**
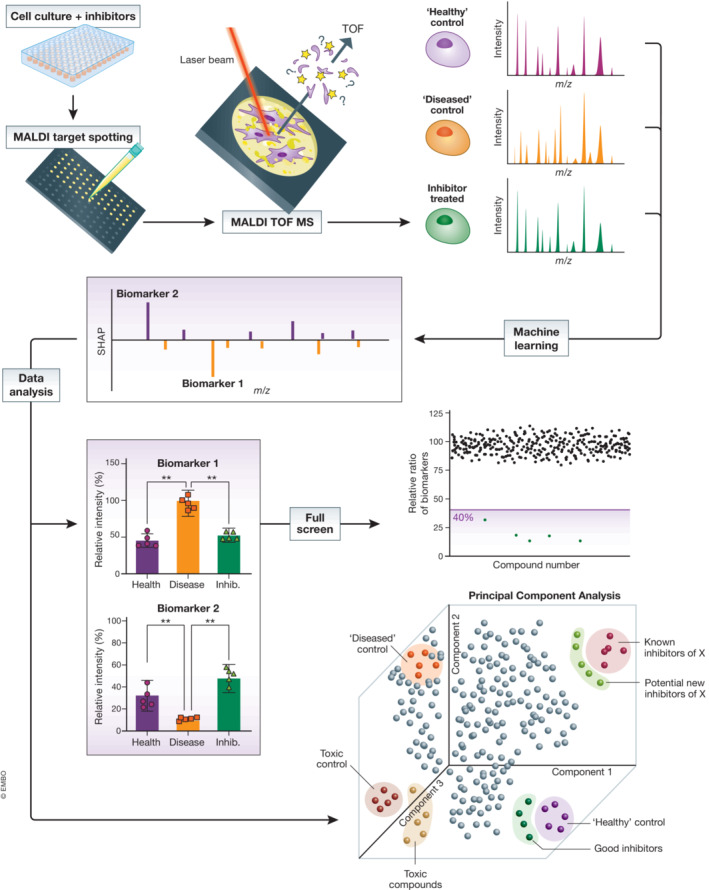
Cellular phenotypic assays by MALDI‐TOF mass spectrometry Cells or extracts of cells are spotted by liquid handling robots onto a MALDI target. MALDI‐TOF MS analysis of these samples defines “fingerprints” of “healthy” and “diseased” controls. Characterization of these complex fingerprints, potentially through machine learning or dimensionality reduction analysis (such as principal component analysis), allows the identification of biomarkers specific for the phenotypes. Changes of these biomarkers can be used as a read‐out for a drug discovery screen against many chemical moieties. Since the readout produces information‐rich multi‐dimensional data, the use of known inhibitors and cytotoxic compounds can be used to multiplex and identify novel compounds in these pathways.

For mammalian cells, both the low‐molecular mass range (100–1,000 Da), which is mostly populated by lipids and high‐abundant cellular metabolites, and the high molecular range (2,000–20,000 Da), which contains peptides and small proteins, can be used for fingerprinting. Fingerprinting of mammalian cell protein biomarkers has been successfully applied to phenotype different cancer cell lines (Serafim *et al*, 2017), classify immune cells (Ouedraogo *et al*, 2012), iPSC embryonic stem cell differentiation (Heap *et al*, 2019), as well as monitor early stress or apoptosis signals in cell lines (Schwamb *et al*, 2013). The classification of cell lines from primary tissues can be complicated by cell heterogeneity, but MALDI‐TOF MS has proven to be sensitive and robust at distinguishing tissue‐derived cell mixtures (Petukhova *et al*, 2019), as well as classifying differentiated cells from primary blood monocytes (Portevin *et al*, 2015). Typically, these strategies use multivariate analysis and identification of unique features for classification that, when combined with flow cytometry, microscopy or known biomarker analysis, result in robust MALDI‐TOF MS methodologies. A proposed strategy to perform an intact cell HT assay using MALDI‐TOF MS is depicted in Fig 4. Cells grown with a “diseased” state and a “healthy” control state are cultured with relevant control compounds and a library of compounds to be tested. The metabolite profiling of these cells using MALDI‐TOF MS will provide a number of biomolecules (i.e., biomarkers) representing the “diseased” and “healthy” cellular states. Relevant biomarkers can be used as a read‐out for HTS using their ratio in the whole data set. Alternatively, unsupervised approaches or machine learning strategies can provide multidimensional insights if compounds return the “diseased” state back to “healthy.” If additional, known inhibitors of relevant pathways or inducers of cell toxicity are added, the assay can be multiplexed to obtain additional information on the compounds tested.

In the low‐molecular range, mammalian cells exhibit dynamic lipid profiles that are often indicative of cell phenotype or disease state (Reddy & Sambasiva Rao, 2006; Fuchs & Schiller, 2008; Goldberg Ira *et al*, 2012). Imaging mass spectrometry (IMS) has already demonstrated that MALDI‐MS is well suited for lipid analysis of cells, and therefore MS imaging methods for lipids have been expanded into cellular classification (Holčapek *et al*, 2015). These assays are exceptionally sensitive, requiring small numbers of cells or even allowed the profiling of single cells such as the classification of astrocytes and neurons by Neumann *et al* (2019a, 2019b), who were also able to show that this was robust across 30,000 individual rodent cerebellar cells. In another study, a proof‐of‐concept assay demonstrated that inhibitors of fatty acid synthase (FASN), which are key for cancer proliferation can be identified by MALDI‐MS (Weigt *et al*, 2019). Combined with automated liquid handling and sample preparation, this study demonstrates that lipid analysis of whole mammalian cells is suitable for development of drug discovery assays to identify inhibitors of lipid metabolism (Weigt *et al*, 2018).

More recently, an automated, label‐free MALDI‐TOF MS cell assay was developed to measure compound uptake and inhibition of that uptake through the transporter OATP2B1 (Unger *et al*, 2020, 2021), providing a proof of principle for the application of MALDI MS cellular assay for rapid direct assessment of drug transporter function. Here, a 384‐well plate was prepared in less 2 min and analyzed in 10 min. In another study, a label‐free cellular phenotypic drug discovery assay was developed to identify anti‐inflammatory drugs in human monocytes derived from acute myeloid leukaemia. The screen identified that the inhibitor nilotinib blocked LPS‐induced inflammatory responses (preprint: Marín‐Rubio *et al*, 2021).

Even though IMS is mainly used to visualize the localization of compounds in tissue sections, the instrumentation also lends itself well to microarray formats, which can increase sample throughput. These microarray formats can be used for tissue or organ sections as these grid‐like applications generates defined coordinates for systematic sampling across a surface (Groseclose *et al*, 2008). In a recent attempt to incorporate cell‐based assays using MALDI‐IMS, Guevara *et al* (2021) integrated microarrays and MALDI‐IMS to demonstrate the potential for miniaturized (down to 40 nl and 10 cells per spot) HT cell screening with the biochemical analysis capabilities of MS in a single platform.

## Limitations, future outlook, and conclusions

Advancement in the instrumentation and methods have led to the increasingly widespread acceptance and utilization of MS‐based HTS platforms in drug discovery R&D. This has been particularly impactful in the HTS field where MS assays have typically lacked the throughput to compete with conventional fluorescence or luminescence assays. An ideal MS‐based HTS platform can now meet the following criteria: speed of analysis, robustness, low sample volume, high sensitivity, ease of use, wide mass range coverage, accurate and precise quantification without the need for compromises in assay design, and direct detection of native biological analytes. In the past decade, there has been a surge in the development of these assays covering a wide variety of MS techniques and biological targets covered in this review. MS‐based HTS approaches are continuously improving in terms of throughput and sensitivity; this is offset by certain limitations discussed herein. MS is often not compatible with biochemical assay reactions that contain high concentration of salts, detergents and common buffering agents as these can induce ion suppression and poor assay robustness and reproducibility (Chandler *et al*, 2016; Belov *et al*, 2020). This is particularly inherent to MALDI where poor spot‐to‐spot reproducibility can occur, and a highly variable response is observed as a consequence. This is also common in AMI/ADE approaches that require careful consideration of assay volumes, buffers, and solvents to ensure uniform ejection and ionization. The most appropriate MS technique for tackling non‐suitable assay compositions is often RapidFire‐MS. However, this is one of the lowest throughput technologies available for developing HTS MS assays. These problems can be mitigated through a more judicious selection of assay matrix components, by applying an appropriate internal standard, performing an on‐target washing step, or by conducting relative quantification by measuring substrate‐to‐product ratio. Moreover, MS‐based readouts are susceptible to isobaric interference, which can be a source of false results for analytes within the mass range of the test compounds. To alleviate this issue, Winter *et al* (2022) suggest using counter assays (tandem MS or orthogonal readout technologies) to rule out false positives.

Further method validation and multi‐site standardization of sample preparation, data acquisition and data processing strategies will be needed to define best practices and reporting guidelines. There have been considerable efforts in the last two decades to address the crisis of reproducibility for drug discovery by incorporating the best practise in assay methodologies. The recommendation put forth in the National Center for Advancing Translational Sciences Assay Guidance Manual, a guide originally developed by Eli Lilly, is a great resource that offers step‐by‐step guidance for drug developers for planning and creating projects for HTS, as well as other steps in the drug discovery pipeline (Markossian *et al*, 2004, 2021). This resource is updated quarterly with more than 100 authors' contributions to date.

Alternative hit‐identification strategies such as ASMS, covalent FBDD and phenotypic assays have also benefited greatly from the recent advancements in MS technologies. For ASMS and covalent fragment screening in particular, MS underpins the screening concept and is critical for identification and characterization of positive binders. The throughput of LC–MS ASMS now rivals that of traditional HTS for hit‐ID and in more recent years novel strategies and platforms such as MALDI/SAMDI‐TOF‐MS have also demonstrated promise. For covalent fragment drug discovery, MS plays an important role in both initial screening, subsequent characterization of binding, as well as chemoproteomics to understand MoA and target engagement in a biological system. These approaches do not necessarily fit HT criteria as of yet, with sample preparation and instrument limitations often being a bottleneck. The further development of novel, state‐of‐the‐art MS platforms will likely factor as to whether these alternative drug discovery approaches to classical hit ID campaign can be incorporated into routine discovery screening strategies.

## Author contributions

**Maria Emilia Dueñas:** Conceptualization; investigation; writing – original draft; writing – review and editing. **Rachel E Peltier‐Heap:** Conceptualization; investigation; writing – original draft; writing – review and editing. **Melanie Leveridge:** Conceptualization; writing – original draft; writing – review and editing. **Roland S Annan:** Conceptualization; supervision; writing – original draft; project administration; writing – review and editing. **Frank H Büttner:** Conceptualization; writing – original draft; writing – review and editing. **Matthias Trost:** Conceptualization; supervision; writing – original draft; project administration; writing – review and editing.

## Disclosure and competing interests statement

REP‐H, ML, and RSA are employees of GSK. FHB is an employee of Boehringer Ingelheim. The other authors declare that they have no conflict of interest.
